# This Place Looks Familiar—How Navigators Distinguish Places with Ambiguous Landmark Objects When Learning Novel Routes

**DOI:** 10.3389/fpsyg.2015.01936

**Published:** 2015-12-24

**Authors:** Marianne Strickrodt, Mary O'Malley, Jan M. Wiener

**Affiliations:** ^1^Max Planck Institute for Biological CyberneticsTuebingen, Germany; ^2^Department of Psychology, Experimental Psychology and Cognitive Science, Justus Liebig University GiessenGiessen, Germany; ^3^Department of Psychology, Bournemouth UniversityPoole, UK

**Keywords:** navigation, spatial cognition, landmark, route learning, ambiguous landmark information

## Abstract

We present two experiments investigating how navigators deal with ambiguous landmark information when learning unfamiliar routes. In the experiments we presented landmark objects repeatedly along a route, which allowed us to manipulate how informative single landmarks were (1) about the navigators' location along the route and (2) about the action navigators had to take at that location. Experiment 1 demonstrated that reducing location informativeness alone did not affect route learning performance. While reducing both location and action informativeness led to decreased route learning performance, participants still performed well above chance level. This demonstrates that they used other information than just the identity of landmark objects at their current position to disambiguate their location along the route. To investigate how navigators distinguish between visually identical intersections, we systematically manipulated the identity of landmark objects and the actions required at preceding intersections in Experiment 2. Results suggest that the direction of turn at the preceding intersections was sufficient to tell two otherwise identical intersections apart. Together, results from Experiments 1 and 2 suggest that route knowledge is more complex than simple stimulus-response associations and that neighboring places are tightly linked. These links not only encompass sequence information but also directional information which is used to identify the correct direction of travel at subsequent locations, but can also be used for self-localization.

## Introduction

Imagine the following scenario: You are sitting on a bus on your way home from work, a route that you have traveled countless times, reading a book. To avoid missing your stop you look out of the window every so often. You see a landmark that you recognize and that you use to localize yourself. However, sometimes you will find that you have judged your location incorrectly, particularly when traveling through environments with few distinctive landmarks, or environments that feature several similar environmental cues. In such situations the current view of an environment may not be sufficient to identify the current location along the route. Instead, further landmark information or experiencing specific changes in movement direction are needed to inform about the actual location along the route. This scenario highlights questions that have rarely been addressed in navigation research: How do navigators deal with visually similar spatial situations when learning novel routes? And, what information do they use to disambiguate these situations during travel? In this study we address these questions by systematically manipulating landmark informativeness (Stankiewicz and Kalia, 2007), i.e., how informative a landmark is about the navigator's location along the route and the action to take. We will argue that knowledge about the sequence of turns and the order of landmarks encountered along the route is an integral part of route knowledge that (1) provides navigators with information about their current location, i.e., serial position, along the route and (2) that is used to disambiguate similar spatial situations.

Route navigation—i.e., following a known path between two locations in a large environmental scale space (Wiener et al., 2009)—belongs to the most common human navigation tasks. While successful route following can be based on different strategies, navigators always need to memorize and recall the direction in which a route continues at several decision points. The nature of the underlying memory depends on the environment (Waller and Lippa, 2007; Chan et al., 2012): In environments without environmental cues or landmarks that allow distinguishing between decision points, navigators have to memorize and recall a sequence of turns and distances. If, on the other hand, the environment features landmarks, navigators typically rely on stimulus-response strategies in which the recognition of a landmark triggers an action (Trullier et al., 1997). In case landmarks are positioned such that a movement toward them brings the navigator closer to the destination (“Go toward the church”), they serve as beacons and recognition of relevant landmarks is sufficient to inform navigators about the direction of travel along the route (Waller and Lippa, 2007). Most route learning models, however, state that landmarks function as associative cues for actions (Siegel and White, 1975; O'Keefe and Nadel, 1978). During route learning landmarks become associated with a motor responses that are defined relative to the body axis (“Turn right at church”) (Wolbers and Wiener, 2014). Thus, in contrast to beacon-based strategies, associative cue strategies require the explicit encoding of directional information. Comparisons of route learning performance in different environments have demonstrated that route learning with landmarks is faster and reaches better overall performance than route learning without landmarks. Moreover, route learning in environments that allow for more parsimonious beacon-based strategies is superior to route learning in environments that require associative cue strategies (Waller and Lippa, 2007; Wiener et al., 2013).

How do we select landmarks during route learning? Not all environmental cues make good landmarks. When learning routes, navigators parse the route into segments (Allen, 1981; Berendt and Jansen-Osmann, 1997), they differentiate between high- and low-information segments (Allen et al., 1979), pay more attention to high-information segments (Allen and Kirasic, 2003), and they remember environmental cues located at decision points more reliably and faster than those located between decision points (Aginsky et al., 1997; Janzen, 2006). Moreover, when presented with the objects that navigators encountered during route learning, neuronal activity in the parahippocampal gyrus is modulated by the navigational relevance of these objects. Specifically, activation was stronger for objects at decision points that serve as associative cues (i.e., landmarks) for route knowledge than for objects that were not at decision points, suggesting a selective neural representation for landmarks (Janzen and van Turennout, 2004; Janzen and Weststeijn, 2007; Schinazi and Epstein, 2010).

What other object properties make for good landmarks? Landmarks are typically thought of as distinctive and identifiable elements or objects in the environment that serve as reference points and allow identifying specific locations during navigation (Lynch, 1960; Siegel and White, 1975; Presson and Montello, 1988; Caduff and Timpf, 2008). Stankiewicz and Kalia (2007)—in line with other landmark models—highlight three main properties of landmarks: (1) *Persistence*, that is a landmark should be present when navigators return to the same location; (2) *Salience*, that is landmarks should be distinctive and easily identifiable when navigators return to the same location; (3) *Informativeness*, that is landmarks need to provide navigators with information relevant to the navigation task. In the context of this study, it is important to point out that informativeness has at least two dimensions: First, landmarks can be informative about the navigator's location, i.e., their serial position, along the route and second, landmarks can be informative about the action navigators have to perform when encountering the landmarks (Stankiewicz and Kalia, 2007).

Let us now consider landmark *informativeness* in the context of route navigation and in the context of the scenario we have outlined at the beginning of the introduction: if all landmarks along a route are unique and used as associative cues or as beacons they are both informative about the navigator's location along the route and informative about the action the navigator has to perform (e.g., “Turn left at church”). However, if visually similar of even identical environmental cues are selected as landmarks the situation becomes more difficult. The non-unique landmark objects by themselves do not allow unambiguously identifying the navigator's location along the route. During route navigation, however, this may not be problematic as long as the same action is associated with all non-unique landmark objects (e.g., “Turn left whenever encountering a church”) as these non-unique landmarks are still informative about the action to perform. If, in contrast, different actions are associated with the non-unique landmark objects (“Turn left at first church”, “Turn right at second church”), the landmark objects by themselves are neither informative about place nor action.

So, how do navigators deal with non-unique landmarks, how do they disambiguate visually similar decision points, and what happens if identical landmarks are associated with turns in different directions? To our knowledge only one study so far has addressed this question (Janzen and Jansen, 2010). In this functional brain imaging study, participants first learned a long route featuring unique and non-unique landmarks. The test phase was comprised of a recognition test during which participants were presented with landmark objects as well as novel objects. Increased activity in the right middle frontal gyrus was recorded for non-unique landmark objects that were associated with different movement directions. Janzen and Jansen (2010) related this increased activation to higher cognitive functions that are required when dealing with ambiguous landmark information. As route learning performance was not reported and as the recognition task did not assess knowledge about the actual actions associated with the landmark objects, our understanding of the behavioral mechanisms involved in learning routes with non-unique landmark objects is still limited.

In the following, we present two experiments that addressed how navigators deal with non-unique landmark objects. In Experiment 1 we systematically manipulated both dimensions of landmark informativeness (location and action) to investigate their effect on route learning. In Experiment 2, we studied what information navigators use to disambiguate decision points along a route that feature identical landmark objects.

## Experiment 1

The primary aim of Experiment 1 was to compare how manipulating both dimensions of landmark informativeness (Stankiewicz and Kalia, 2007)—i.e., location and action—affect the learning of a novel route. First, we manipulated the uniqueness of landmark objects, therefore manipulating how informative the landmark object is about the navigator's location, i.e., the serial position along the route. Half of the landmark objects appeared only once in the environment, therefore uniquely identifying the intersection they were associated with (*unique intersections*). The other half of the landmark objects appeared twice in the environment, i.e., they were associated with two intersections (*non-unique intersections*). Thus, landmark objects at non-unique intersections by themselves did not allow for the identification of the associated intersection unambiguously. Second, we manipulated how informative landmark objects were by manipulating the actions navigators had to perform when encountering them. Specifically, landmark objects that appeared twice in the environment (non-unique landmark objects) were either associated with the same movement direction (e.g., “Turn left at both churches”), or were associated with different movement direction (e.g., the route would take a left turn at the first church and a right turn at the second church). The manipulations of landmark informativeness are summarized in Table 1.

**Table 1:** **Manipulation of the two dimensions location and action of landmark informativeness (Stankiewicz and Kalia, 2007) at the three types of intersections used in Experiment 1**.

**Type of intersection**	**Informativeness**	
	**Location**	**Action**
Unique	High	High
Non-unique—same direction	Low	High
Non-unique—different direction	Low	Low

### Predictions

We expect that these manipulations affect route learning as follows. Unique intersections containing landmark objects only presented once along the route are informative about location (as they appear only once in the environment resulting in high location informativeness) as well as action (unique S-R associations, resulting in high action informativeness) and should therefore yield good learning performance. Non-unique landmark objects presented at two intersections and associated with the same movement direction have a reduced location informativeness (as two intersection cannot be distinguished on basis of the landmark object alone), but have a high action informativeness (as the same action has to be taken at both intersections with the same landmark objects). In case route learners rely on simple S-R associations (Waller and Lippa, 2007), these intersections should not lead to decreases in learning performance as compared to unique intersections, even though location informativeness is lower than for unique intersection. Indeed they may result in increased performance as the same S-R association is encountered twice along the route. Finally, non-unique landmark objects associated with different movement direction have reduced location informativeness as well as reduced action informativeness (as different action has to be taken at both intersections with the same landmark objects). Performance in learning these intersections should be affected most, as decisions cannot be solely based on the information available at the intersection.

### Methods

#### Participants

Thirty-eight Psychology undergraduates with normal or corrected-to-normal vision participated in the study and were awarded course credits. Two participants were removed from the final data set as their performance for intersections with unique landmarks did not exceed chance level performance (33%) in the last experimental session. Of the remaining 36 participants, 18 were females and 18 were males. As this study was not designed to investigate gender differences and as exploratory analyses did not reveal any gender effects in either Experiment 1 or Experiment 2, gender is not reported in the further analysis. Their average age was 21.36 years old (*SD* = 5.42). The experiment was approved by the Bournemouth University Ethics Committee. All participants were fully informed of the nature of the experiment and gave written informed consent.

#### Materials

**Figure 1:**
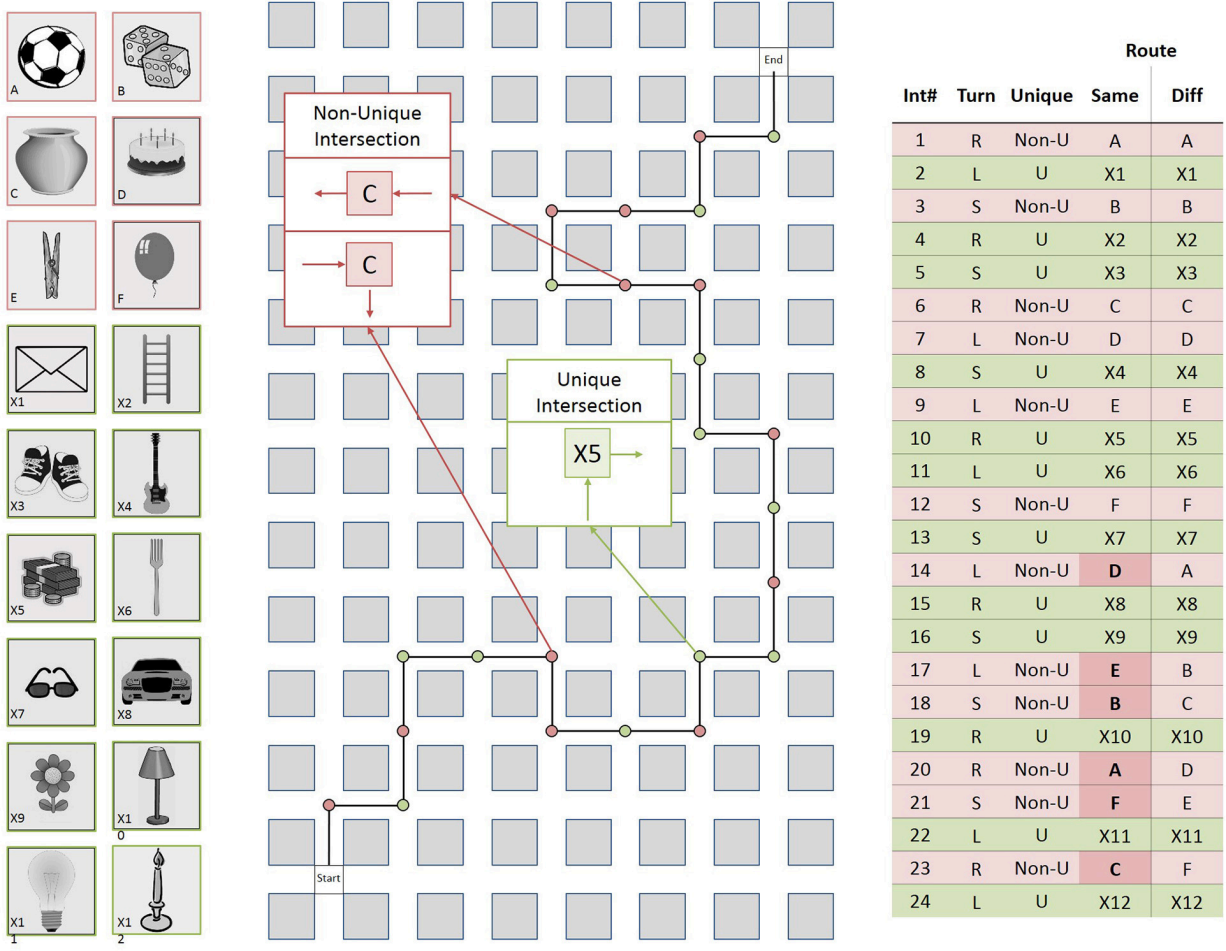
**Left:** The 18 landmark objects used in the routes; **Middle:** Schematic drawing of the *different direction route*; **Right:** Table summarizing the *same direction* and the *different direction* route in detail. Int#: Number of intersection; Turn: Direction of turn left (L), right (R), straight (S); Unique: Unique (U), or non-unique (Non-U) object at intersection; Route: Two route conditions; Same: Direction of turn is the same for pairs of non-unique intersections; Diff: Direction of turn is different for pairs of non-unique intersections.

**Figure 2:**
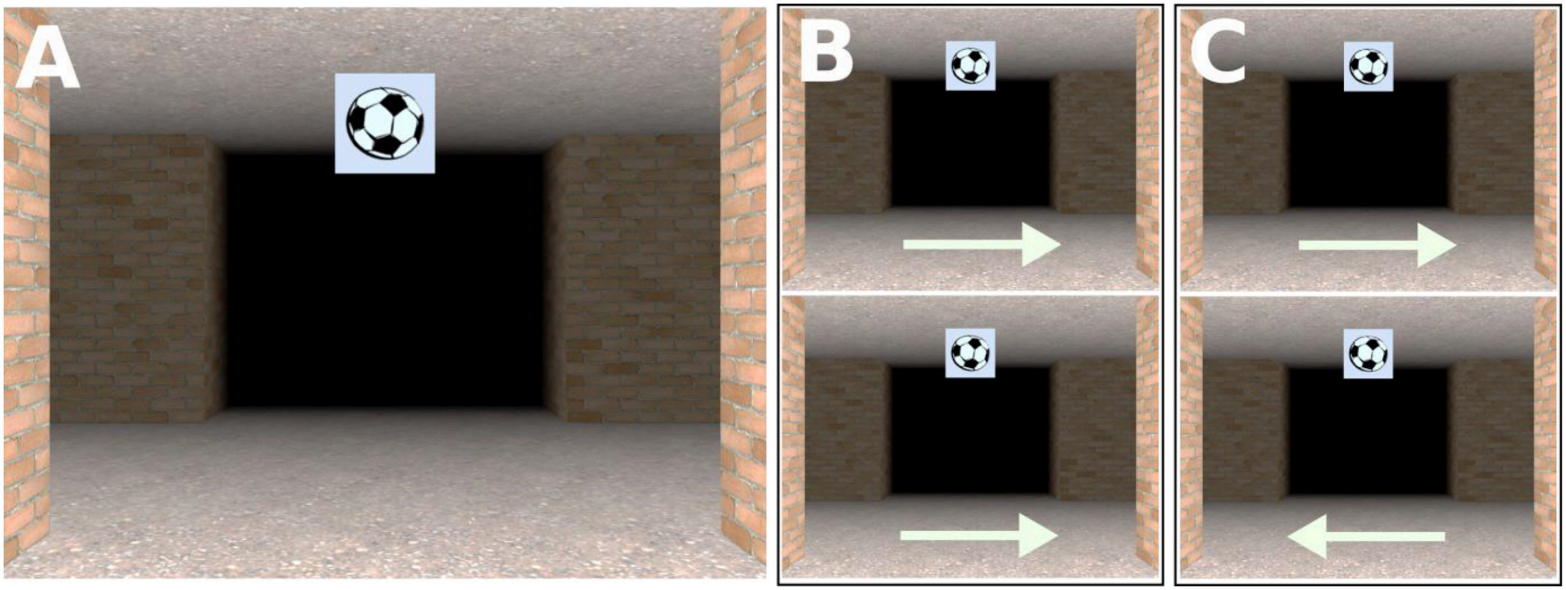
**(A)** Screenshot taken at an intersection. The landmark object associated with that intersection is the picture of a football suspended from the ceiling; **(B)** Non-unique same direction intersections; the route continues in the same direction at the intersections with the same landmark object; **(C)** Non-unique different direction intersection; the route continues in the different direction at the intersections with the same landmark object.

Using Vizard 3.0 (WorldViz), we created two virtual routes each consisting of 24 four-way intersections connected by corridors. Each corridor was 20 m long, 5 m wide and had a height of 4 m, rendering the length of the entire route 500 m. Each intersection featured one image of an object (1 × 1 m) that was mapped onto a cube suspended from the ceiling (see Figures 1, 2). To prevent participants from seeing more than one intersection at a time, we introduced black fog at a distance of 12.7 m from the virtual camera which was moved through the virtual environment at a height of 1.8 m. The experiment was displayed on a 21.5” widescreen monitor with a resolution of 1920 × 1080 pixels.

For each route, 18 objects were used to create two types of intersections: twelve objects appeared at only one intersection each, thus creating twelve unique intersections; each of the remaining six objects appeared at two intersections along the route, thus creating six pairs of non-unique intersections. On both routes, there were six unique and six non-unique intersections in the first half of the route and six unique and six non-unique intersections in the second half of the route and the repeated landmark objects were distributed about both halves of the route. Navigation along the routes required right turns at eight intersections, left turns at eight intersections, and going straight at eight intersections. The number of right turns, left turns, and straights were balanced across types of intersections.

In the *same direction route*, the direction of turn was always the same for pairs of non-unique intersections. For example, if the route turned left at the first intersection featuring a church it would also turn left at the second intersection featuring a church. In contrast, in the *different direction route*, the direction of turn was different for pairs of non-unique intersections. In this case, if the route turned left at the first intersection featuring a church, it would turn right or go straight at the second intersection featuring a church. Neither the *same direction* nor the *different direction* route crossed itself. In order to analyse the impact of the *intersection position* along the route on route learning performance (Allen, 2000), the unique and non-unique intersections were labeled independently as belonging either to the initial portion (i.e., the first four unique intersections and first four non-unique intersections along the route), the middle portion (positions five to eight), or the final portion (positions nine to twelve) of the route.

#### Procedure

Participants were randomly assigned to either the *same direction route* or the *different direction route*, rendering the turning direction at pairs of non-unique intersection the between-subject factor. The experiment consisted of six experimental sessions. Each session consisted of a training phase and a test phase. In the training phase, participants were passively transported along the entire route at a speed of 3 m/s and instructed to memorize the route. During this training phase they therefore experienced both translations between intersections and the turns at intersections. A single training run took 155 s. In the test phase, participants were transported toward all 24 intersections in the same order as during training. However, in contrast to the training phase, participants would be transported to the middle of each intersection and asked to indicate the direction in which the routes continued by pressing the “left,” “right,” or “up” arrow key as soon as they were confident they knew the correct answer, but within five seconds after movement stopped. A button press would then initiate the next trial, starting the auto-pilot in the middle of the next corridor. Hence, no feedback about the correct direction of turn was provided during the test phase.

### Results

**Figure 3:**
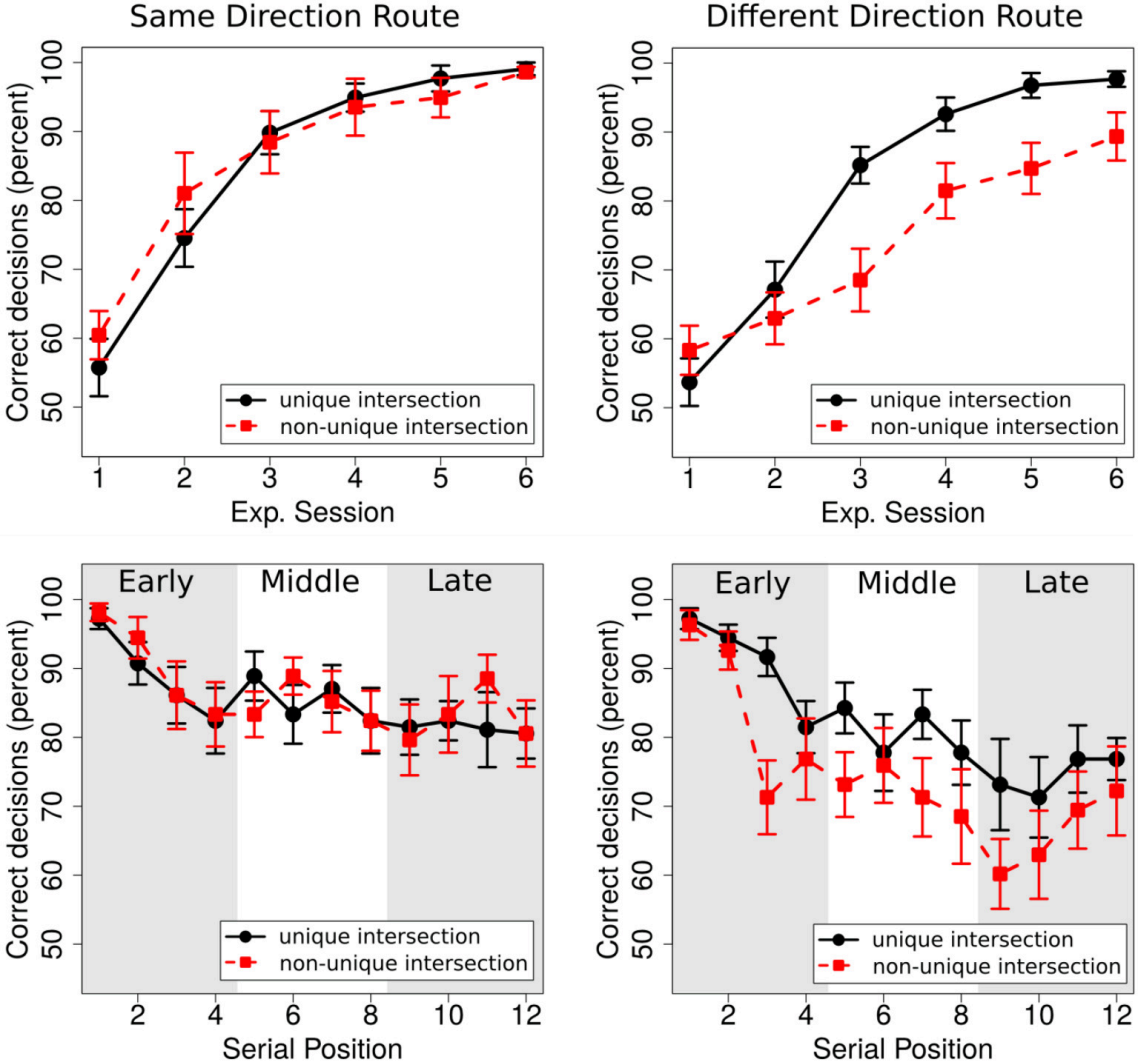
**Top row:** Performance for unique and non-unique intersection in the same direction route (left) and the different direction route (right). **Lower row:** Performance as a function of serial position for the same direction route (left) and the different direction route (right). For the analysis, intersections were classified as early, middle, or late along the route.

We ran a 2 × 2 × 3 × 6 repeated measures ANOVA with the between factor *route* (same direction, different direction) and the within factors *uniqueness* of landmark (unique, non-unique), *experimental session* (1–6), and *portion* (initial, middle, final). Given that multiway ANOVAs harbor a multiple comparison problem, we used sequential Bonferroni corrections to control for familywise error rate (Cramer et al., 2015). We only report effects that survived this correction. The ANOVA revealed significant main effects of *uniqueness* [*F*_(1, 34)_ = 8.24, *p* < 0.01, $\eta_{p}^{2}=0.20$], *experimental session* [*F*_(5, 170)_ = 97.69, *p* < 0.001, $\eta_{p}^{2}=0.74$], and *portion* [*F*_(2, 68)_ = 23.53, *p* < 0.001, $\eta_{p}^{2}=0.41$]. Specifically, overall performance was better *for unique intersections* (83.75 ± 1.32% [SE]) than for *non-unique intersections* (80.22 ± 2.30% [SE]), and performance for both unique and non-unique intersections was significantly above chance level [33%; unique intersections: *t*_(35)_ = 38.31; *p* < 0.001; non-unique intersections: *t*_(35)_ = 20.40; *p* < 0.001]. Performance increased over *experimental sessions* (from 57.05 ± 2.10% [SE] in session 1 to 96.18 ± 1.21% [SE] in session 6, see Figure 3), and performance decreased over *the portion of the route* (initial portion: 88.77 ±1.34% [SE], middle portion: 80.84 ± 2.02% [SE]; final portion: 76.32 ± 2.59% [SE]; all *post-hoc* comparisons were significant [all *p* < 0.01]).

Three interactions rendered significant results: *route* × *uniqueness* [*F*_(1, 34)_ = 12.49; *p* = 0.001, $\eta_{p}^{2}=0.27$], *session* × *uniqueness* [*F*_(5, 170)_ = 6.87, *p* < 0.001, $\eta_{p}^{2}=0.17$], and *session* × *portion* [*F*_(10, 340)_ = 4.73, *p* < 0.001, $\eta_{p}^{2}=0.12$].

For the current study, the interactions involving uniqueness are of particular interest as they suggest that performance for unique and non-unique intersections was affected differently in the two routes and over experimental sessions. To further investigate the nature of these effects, we conducted two separate ANOVAs, one for each of the two routes (same-direction, different-direction), with *uniqueness* (unique, non-unique) and *experimental session* (1–6) as within factors. For the *same direction route*, the ANOVA revealed a main effect of *session* [*F*_(5, 85)_ = 43.73, *p* < 0.001, $\eta_{p}^{2}=0.72$], but no main effect of *uniqueness* [*F*_(1, 17)_ = 0.20, *p* = 0.66, $\eta_{p}^{2}=0.01$] and no significant interaction [*F*_(5, 85)_ = 1.73, *p* = 0.14, $\eta_{p}^{2}=0.09$]. For the *different direction route*, in contrast, the ANOVA revealed main effects for *session* [*F*_(5, 85)_ = 57.07, *p* < 0.001, $\eta_{p}^{2}=0.77$] and *uniqueness* [*F*_(1, 17)_ = 24.46, *p* < 0.001, $\eta_{p}^{2}=0.59$], as well as a significant interaction [*F*_(5, 85)_ = 6.85, *p* < 0.001, $\eta_{p}^{2}=0.29$]. *Post-hoc* tests revealed significant differences between unique and non-unique intersections for experimental sessions 3–6 (all *p* < 0.01), but not for sessions 1 and 2. The most important result of these additional analyses is that uniqueness affects performance only in the *different direction condition*, i.e., when one landmark object is associated with different movement directions. In the *same direction condition*, where one landmark object is associated twice with the same movement direction, on the other hand, landmark uniqueness does not affect performance.

The interaction between *session* and *portion* results from better performance for the initial portion compared to the middle and final portion at the beginning of the experiment as compared to the end of the experiment. Pairwise comparisons reveal significant differences between the initial portion and both the middle and the final portion in Session 1 (early vs. middle: *p* < 0.001; early vs. late: *p* = 0.01; middle vs. late: *p* = 0.52). These differences are not observed in Session 6 (all *p* > 0.05).

### Discussion

The purpose of Experiment 1 was to investigate how the two dimensions of landmark informativeness—i.e., localization and action (Stankiewicz and Kalia, 2007)—affect route learning. Specifically, we compared learning performance for different types of intersections: First, *unique* intersections, i.e., intersections with unique landmark objects that were informative about both, the navigator's location along the route and the action navigators need to take at the associated intersection. Second, *non-unique same direction* intersections that had reduced location informativeness, but were still informative about the action that had to be taken. And third, *non-unique different direction* intersections, that had both, reduced location and reduced action informativeness.

Performance at *unique* intersections and *non-unique* intersection in the *same direction route* was virtually identical. This result demonstrates that manipulations of position informativeness alone did not affect route learning performance, as long as the landmarks were informative about the action navigators had to take. This result is in line with stimulus-response or associative cue strategies in which landmarks are associated with actions or motor responses (Siegel and White, 1975; O'Keefe and Nadel, 1978; Waller and Lippa, 2007). If anything, one could have expected better performance for *non-unique* intersection in the same direction route than for *unique* intersections as the same stimulus-response association was encountered twice along the route.

Performance for *non-unique* intersections in the *different direction* route was worse than for *non-unique* intersections in the *same direction* route, demonstrating that reducing action informativeness did affect route learning performance. Again, this result appears to be in line with stimulus-response theories of route learning, as the same stimulus is associated with different responses (“Turn left/right at church”). Note, however, that performance for all types of intersections clearly exceeded chance level. This is difficult to explain if participants only associated the landmark object at the intersection with an action, as it would not allow distinguishing between *non-unique* intersections that required different actions (i.e., *non-unique* intersection on the *different direction* route). This raises two questions: First, what information did participants use to distinguish between intersections with identical landmark objects? Second, performance exceeded chance level for all types of intersection, demonstrating that participants were able to distinguish between intersections with identical landmark objects. So why was their performance impaired for *non-unique* intersections that required different actions as compared to unique intersections or non-unique intersections that required the same action? The first question will be addressed in Experiment 2, and we will return to the second question in the general discussion.

Performance in the initial portion of both routes was higher than in the middle or final portion of the route (cf. Allen, 2000). This effect disappeared in later experimental sessions, i.e., when participants have reached overall high performance levels. Serial order effects in route learning highlight the functional importance of the order in which landmark and turning information is encountered along the route (Helstrup and Magnussen, 2001).

## Experiment 2

Experiment 1 demonstrated that participants could distinguish between intersections that featured the same landmark object. The aim of Experiment 2 was to investigate what information participants used to do so. Two possibilities immediately spring to mind: First, in addition to simple S-R associations, i.e., associations between single landmarks and actions, participants label non-unique intersections based on whether it is their first or second encounter with the corresponding landmark (e.g., “Left at first church”, “Right at second church”). Alternatively, participants rely on a more integrated route representation that entails information about the overall sequence of landmarks and the turns along the route. Both alternatives would allow them to track their location along the route in order to distinguish visually identical (non-unique) intersections. While the first explanations appears to be more parsimonious, already early theories of route knowledge (O'Keefe and Nadel, 1978) have suggested stimulus-response-stimulus (S-R-S) type of associations instead of simple S-R associations. Such S-R-S associations not only allow anticipating the next landmark or place along a route (Trullier et al., 1997; Wiener et al., 2012), they also allow embedding single decision points into a route representation that can be used to track progress along the route. So far, empirical evidence for such more integrated route representations comes primarily from priming studies demonstrating that the recognition of a place along a route is facilitated when primed with an image of the preceding place or landmark (Schweizer et al., 1998; Schinazi and Epstein, 2010).

To distinguish between the possible explanations, we created a route that consisted of 12 pairs of *non-unique* intersections. After learning the route, participants were presented with videos of short sections of the route that were presented in randomized order. Each video covered one intersection (the predecessor) and approached the subsequent intersection, where participants were asked to indicate the direction in which the route continued. Given the randomized presentation of the trials in the test phase, the use of simple order information (“Left at first church”, “Right at second church”) to distinguish non-unique intersections would have resulted in chance level performance. An integrated route representation in which neighboring places are tightly linked, however, would allow distinguishing non-unique intersections either (1) on basis of landmark information at the predecessor intersection or (2) on basis of landmark as well as direction information at the predecessor intersection. The design of the test situations—explained in more detail below—allowed us to distinguish between these possibilities.

### Methods

#### Participants

Eighteen Psychology undergraduate students from Bournemouth University with normal or corrected-to-normal vision participated in the study and were awarded course credits. Two participants were removed from the final data set as their performance in the last experimental session did not exceed chance level performance (33%), i.e., they have not learned the route. Of the remaining 16 participants, nine were females and seven were males. Their average age was 20.8 years (*SD* = 2.32). The experiment was approved by the Bournemouth University Ethics Committee. All participants were fully informed of the nature of the experiment and gave written informed consent.

#### Materials

Vizard 3.0 (WorldViz) was used to create a video of a virtual route consisting of 24 intersection. As in the routes of Experiment 1, each intersection featured one image of an object that was mapped onto a cube suspended from the ceiling (see Figure 2). The dimensions of the corridors and landmarks, the camera height, movement speed and fog distance were identical to Experiment 1.

**Figure 4:**
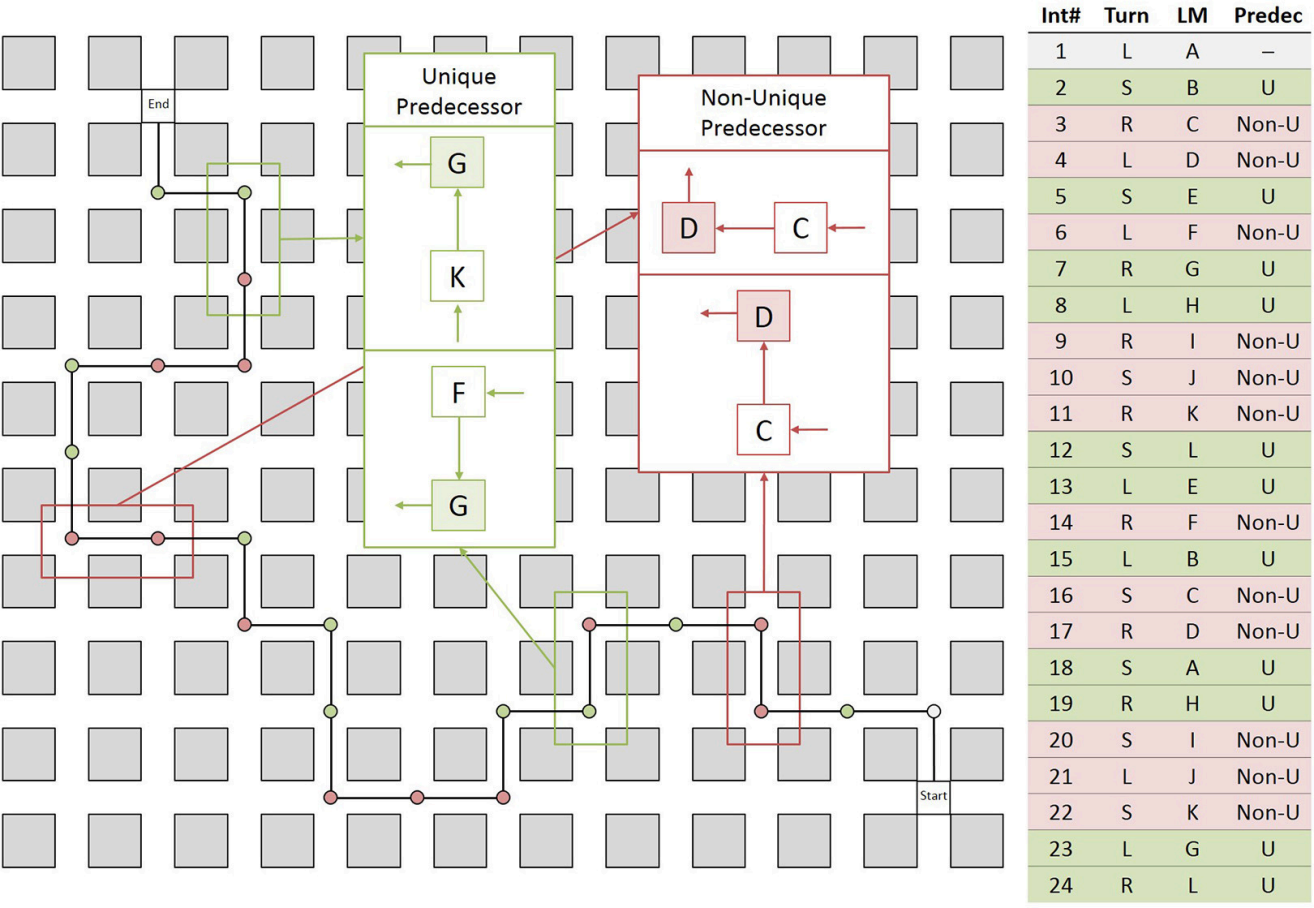
**Left:** Schematic drawing of the route and example of unique predecessor (green) and non-unique predecessor intersections (red). *Unique predecessor intersections* are preceded by intersections with different landmark objects, while non-unique predecessor intersections are preceded by intersections with the same landmark object. The only way to disambiguate the two non-unique predecessor intersections is by taking into account the movement direction at the preceding intersections; **Right:** Table describing the route in detail. Int#: Number of intersection; Turn: Direction of turn left (L), right (R), straight (S); LM: Landmark objects, same letters indicating same object; Predec: Predecessor object unique (U) or non-unique (Non-U).

Twelve objects were used and each object was placed at two intersections, thus creating 12 pairs of intersections that were visually identical. These pairs were distributed along the route such that they formed two different types of intersections: *unique predecessor intersections* and *non-unique predecessor intersections*, which differed with respect to the landmark objects encountered at the preceding intersection. Two intersections with the same landmark object were classed *unique predecessor intersections* if they were preceded by different landmark objects and were classed *non-unique predecessor intersections* if they were preceded by the same landmark object (see Figure 4). Thus, in contrast to Experiment 1, uniqueness was not determined by the presence of another intersection featuring the same landmark object, but by the combination of two landmark objects at neighboring intersections. The direction of turn at intersections featuring the same landmark as well as at their preceding intersections (for both unique and non-unique predecessor intersections) was always different.

Note that *unique predecessor intersections* can be disambiguated solely by the identity of the landmark at the preceding intersection. *Non-unique predecessor intersections*, in contrast, cannot be disambiguated by the identity of the landmark at preceding intersection alone, but require taking into account the movement direction at the preceding intersection (see Figure 4).

The original video was used to create short test videos for the test phase, each covering the navigation along one intersection of the original route and approaching the subsequent intersection. The videos were stopped short of the second intersection, which was either a *unique* or a *non-unique predecessor* intersection. Each intersection along the route, apart from the first intersection, which did not possess a predecessor, was utilized to create a video, adding up to 23 test videos (11 ending at a *unique predecessor* intersection and 12 ending at a *non-unique predecessor* intersection).

#### Procedure

The experiment consisted of a learning phase and a test phase.

##### Learning phase

Participants were shown the video of the entire route and instructed to memorize the route. The video was paused just short of each of the 24 intersections along the route, where participants were asked to indicate the movement direction required to proceed along the route. After the response the video continued, thus providing participants with feedback about the correct direction of turns. Participants' responses were recorded which allowed us to monitor the learning of the route. This learning procedure was repeated six times, before participants entered the test phase.

##### Test phase

In the test phase, participants were shown the 23 short test videos in randomized order. When the videos stopped short of the second intersection, participants were asked to indicate the direction to proceed along the original route. Their responses were recorded and the test phase continued with another short video. Thus, during the test phase participants were not informed about the correct direction of turn. Depending on whether or not the second intersection displayed in the video was a *unique predecessor* intersection or a *non-unique predecessor* intersection, these trials were classed *as unique predecessor* trials or *non-unique predecessor* trials. Participants were not informed about the nature of the test phase before completing the learning phase.

#### Predictions

##### Learning phase

Even though the route in Experiment 2 only featured non-unique landmarks, results from Experiment 1 suggest that that participants are able to learn the route during the learning phase.

##### Test phase

As highlighted in the introduction of Experiment 2, different strategies could have allowed participants to disambiguate non-unique intersection in Experiment 1. Experiment 2 makes different predictions for these strategies: (1) If participants relied on memorizing first and second encounter of non-unique landmarks/objects (e.g., “Left at first church”, “Right at second church”) they should perform at chance level in the test phase as trials were presented in random order. (2) If participants primarily relied on the overall sequence in which they encountered landmarks along the route to monitor progress and to disambiguate intersections with identical landmark objects, they should perform well at *unique predecessor* intersections, but at chance level for *non-unique predecessor* intersections. (3) If participants used route representation that integrates both, overall landmark sequence in conjunction with turning directions, they should perform equally well for both types of target intersections.

### Results

#### Training phase

**Figure 5:**
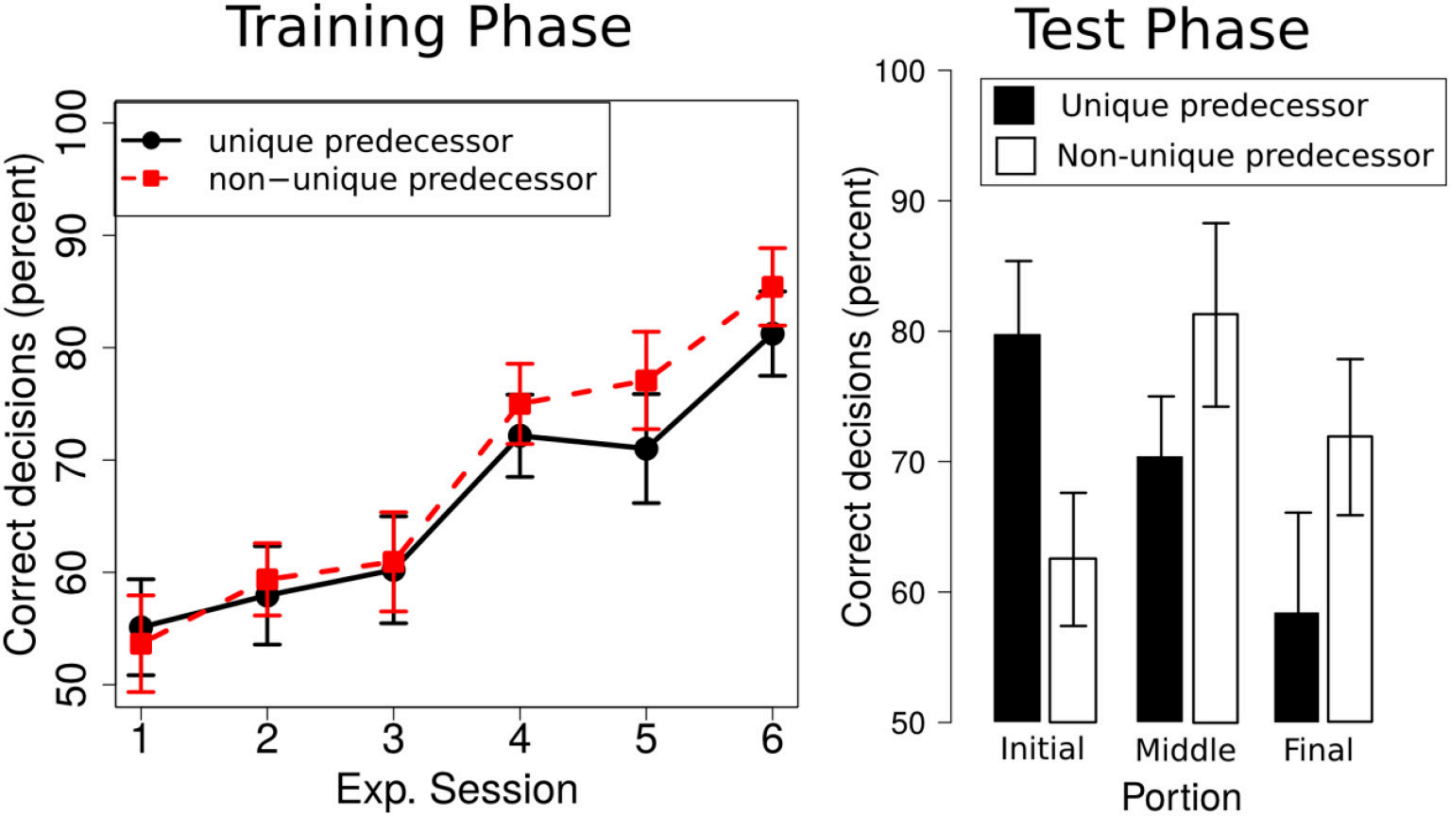
**Left:** Participants' performance in the training phase across the six experimental sessions; **Right:** Performance in the test phase for the *unique predecessor* and the *non-unique predecessor* trials for intersections in the initial, middle or final portion of the route.

We ran a 2 × 3 × 6 repeated measures ANOVA with the within factors *predecessor* (unique, non-unique) and *portion* (initial, middle, final), and *experimental session* (1–6). As in Experiment 1, we used sequential Bonferroni corrections to control for familywise error rate (Cramer et al., 2015). We only report effects that survived this correction. The ANOVA revealed a significant main effect of *experimental session* [*F*_(5, 75)_ = 17.00, *p* < 0.001, $\eta_{p}^{2}=0.53$] and *portion* [*F*_(2, 30)_ = 3.52, *p* = 0.04, $\eta_{p}^{2}=0.19$], but no significant main effect of *predecessor* [*F*_(1, 14)_ = 1.99, *p* = 0.17, $\eta_{p}^{2}=0.11$]. None of the interactions rendered a significant result. Participants' performance increased over experimental session (from 54.35 ± 3.95% [SE] in session 1 to 83.42 ± 2.95% [SE] in session 6, compare Figure 5, left). Overall performance was highest for the initial portion of the route (73.83 ± 4.33% [SE]), followed by the middle portion (65.89 ± 3.20% [SE]) and final portion (62.05 ± 3.90% [SE]). Overall performance for *unique predecessor* intersections was 66.29% (± 3.00% [SE]) and 68.9% (± 2.91% [SE]) for *non-unique predecessor* intersections.

#### Test phase

We ran a repeated measures 2 × 3 ANOVA with the within factors *predecessor* (unique, non-unique) and *portion* (initial, middle, final) which did not reveal significant main effects of *predecessor* [*F*_(1, 15)_ = 0.17, *p* = 0.69, $\eta_{p}^{2}=0.01$] or *portion* [*F*_(2, 30)_ = 1.73, *p* = 0.19, $\eta_{p}^{2}=0.10$], but a significant interaction [*F*_(2, 30)_ = 5.84, *p* < 0.01, $\eta_{p}^{2}=0.28$]. Overall, test phase performance for *unique predecessor* intersections was 70.45% (± 3.57% [SE]) and 71.88% (± 4.74% [SE]) for *non-unique predecessor* intersections. Performance for target intersections in the initial portion of the route was 71.09% (± 3.91% [SE]), 75.78% (± 4.63% [SE]) in the middle portion of the route and 66.07% (± 4.86% [SE]) in the final portion of the route. *Post-hoc t*-tests suggest that the interaction is driven by significantly better performance for unique predecessor trials as compared to non-unique predecessor trials from the first portion of the experiment (*p* = 0.03). In the middle and late portion of the route, performance is numerically higher for non-unique than for unique predecessor trials, but these differences were not significant (both *p* > 0.05, compare Figure 5, right).

Importantly, test phase performance for both unique and non-unique predecessor intersections was significantly above chance level [*t*-test against 33%: unique predecessor intersections: *t*_(15)_ = 19.65, *p* < 0.001; non-unique predecessor intersection: *t*_(15)_ = 15.08, *p* < 0.001]. If we assume that participants have learned during training, that each landmark object was associated with one of only two directions, chance level would increase to 50%. Performance for both unique and non-unique context trials also clearly exceeded this higher and more conservative chance level [*t*-test against 50%: unique predecessor intersections: *t*_(15)_ = 19.60, *p* < 0.001; non-unique predecessor intersections: *t*_(15)_ = 15.05, *p* < 0.001].

### Discussion

Experiment 2 replicated findings from Experiment 1 demonstrating (1) that participants can learn long routes that feature non-unique landmarks and (2) that the initial portion of the route is learned faster than the middle and late portion of the route. In contrast to Experiment 1, however, where repeated landmark objects were isolated, participants encountered the same sequence of up to four consecutive landmark objects twice in Experiment 2 (see Figure 4). While this makes it more difficult to use the sequence in which landmark objects were encountered to disambiguate non-unique intersections, it may not be impossible. Furthermore, participants could still simply memorize whether it was their first or second encounter with a repeated landmark object (“Turn left at first church,” “Turn right at second church”) to disambiguate non-unique intersections during the training session.

In the test phase, in contrast, participants were presented with videos of short sections of the route. Each video showed navigation along one intersection of the original route and approached the subsequent intersection when it was stopped. Participants were then asked to indicate the movement direction to proceed along the original route. As these videos were presented in random order, relying on information such as “Left at first church” and “Right at second church” would have led to chance level performance. Participants, however, performed significantly above chance level, demonstrating that they could utilize information from the preceding intersection to disambiguate their location along the route.

What information did they use? Participants performed above chance level and equally well for *unique predecessor* trials (in which a pair of non-unique intersections were associated with different predecessor landmark objects) and *non-unique predecessor* trials (in which a pair of non-unique intersections were associated with the same predecessor landmark object). These results demonstrate that participants did not solely rely on the overall sequence in which landmarks were encountered along a route to monitor their position and to disambiguate visually identical intersections. If that was the case, we expected chance level performance for *non-unique predecessor* trials. Instead, participants used the turning direction experienced at the last intersection, the predecessor intersection, to disambiguate two visually identical target intersections.

## General discussion

In human navigation, route knowledge is typically conceptualized as based on stimulus response (S-R) associations in which the recognition of a place, view or landmark triggers a movement or motor response, such as turning left or right (Gillner and Mallot, 1998; Mallot and Gillner, 2000; Waller and Lippa, 2007). However, without monitoring progression along the route, simple S-R associations do not allow distinguishing visually similar or identical places that require different responses. To investigate how participants deal with such situations, and what information they use to disambiguate visually similar or even identical places, we created virtual environments that allowed us to systematically manipulate the informativeness of landmark objects that were associated with decision points along the route (Stankiewicz and Kalia, 2007). Collectively, the present experiments illustrate that navigators can distinguish visually identical intersections when learning novel routes (cf. Janzen and Jansen, 2010). In the context of this study, they do so by memorizing the sequence in which landmark objects are encountered along with information about the direction in which the route continues. While further studies are needed to investigate how results from this study translate to real life situations, our finding suggest that route representations are best described as a series of S-R-S associations (O'Keefe and Nadel, 1978). In contrast to the simpler S-R associations, S-R-S associations not only allow predicting the next landmark or place along a route (Trullier et al., 1997; Wiener et al., 2012), they also make ideal building blocks for embedding single decision points into more integrated route representations (Schweizer et al., 1998; Schinazi and Epstein, 2010).

In Experiment 1 we systematically manipulated the two dimensions of the landmark property *informativeness* (Stankiewicz and Kalia, 2007): First, we manipulated *location informativeness*—i.e., how informative single landmark objects were about the navigator's location along the route—by introducing landmark objects that were associated with two rather than just one intersection along the route. Second, we manipulated *action informativeness*—i.e., how informative single landmark objects were about the action to be taken—by manipulating whether or not the same action was required at repeated non-unique landmark objects. Interestingly, the manipulation of *location informativeness* alone, did not affect route learning performance, while the manipulation of *action informativeness* did. In other words, participants' learning performance for non-unique intersections was as good as for unique intersections, as long as the route continued in the same direction at these intersections. Performance for non-unique intersections, however, decreased if these intersections required different actions, i.e., if the route continued in different directions. Despite these performance declines, it is important to note that performance for all conditions or types of intersection was well above chance level, demonstrating that participants were able to distinguish visually identical intersections.

To our knowledge this is the first study that quantified the behavioral consequences of ambiguous landmark information (see Janzen and Jansen, 2010, for a functional brain imaging study). The question why performance declined at visually identical intersections that required different actions, however, is beyond the scope of the current study, but we will briefly outline a number of possible explanations: First, participants used different encoding strategies for unique and non-unique intersections, with additional information required for non-unique, visually identical, intersections. This results in higher memory loads and therefore reduced learning performance. Alternatively, the decreased performance for non-unique intersections could result from the fact that the same stimulus (i.e., landmark object) was associated with different responses (movement directions). The selection of the appropriate response and the suppression of the inappropriate response involve higher levels of cognitive control (Janzen and Jansen, 2010). In other cognitive domains, such response conflicts result in reduced performance (e.g., Botvinick et al., 2001). It is also interesting to note that participants did not perform better for non-unique intersections that required the same response than for unique intersections, even though they encountered the same stimulus-response pairing twice as often. Further research is needed to address these issues in more detail.

Experiment 2 was designed to investigate how participants distinguished visually identical intersections during route learning. Particularly, we asked whether participants encoded order information of pairs of intersections (“Turn left at first church”, “Turn right at second church”), or whether they used knowledge about the preceding intersection to distinguish otherwise identical intersections. To do so, participants first learned a novel route in which each landmark object was associated with two intersections. They were then presented, in randomized order, with short sections of the route covering two intersections and asked to indicate the direction in which the route continued. Participants were able to solve this task, even though they could not rely on order information (“Turn *left* at first church,” “Turn *right* at second church”). Moreover, participants could distinguish pairs of visually identical intersections that were also preceded by visually identical intersections and only differed in the turning direction at the first intersection. This result is particularly impressive as participants were not informed about the nature of the test task when they learned the route.

Taken together, results from Experiments 1 and 2 strongly suggest that route knowledge is more complex than just simple S-R associations. While earlier priming studies have already demonstrated that neighboring places are linked in route knowledge (Schweizer et al., 1998; Janzen and Weststeijn, 2007; Schinazi and Epstein, 2010), our experiment demonstrate that these links not only encompass the sequence in which landmarks are encountered along the route but also directional information. Importantly, this spatial component in route knowledge is not only used to identify the direction of travel at the current location, but can also be used for self-localization.

## Author contributions

MM has been involved in designing the study, data collection, data analysis, and writing the manuscript. MO has been involved in designing the study, data collection, and writing the manuscript. JW has been involved in designing the study, data analysis, and writing the manuscript.

### Conflict of interest statement

The authors declare that the research was conducted in the absence of any commercial or financial relationships that could be construed as a potential conflict of interest.
